# Imaging of rectal cancer

**DOI:** 10.1007/s00117-019-0579-5

**Published:** 2019-10-15

**Authors:** J. Boot, F. Gomez-Munoz, R. G. H. Beets-Tan

**Affiliations:** 1 Department of Radiology, The Netherlands Cancer Institute, Amsterdam, The Netherlands; 2 GROW School for Oncology and Developmental Biology, Maastricht University Medical Center, Maastricht, The Netherlands; 3 Department of Interventional Radiology, Hospital Clinic Universitari, Barcelona, Spain; 4 University of Southern Denmark, Odense, Denmark

**Keywords:** Colorectal carcinoma, Staging, Magnetic resonance imaging, Endorectal sonography, Endoscopic ultrasound, Kolorektales Karzinom, TumorStaging, Magnetresonanztomographie, Endorektale Sonographie, Endoskopischer Ultraschall

## Abstract

International guidelines dictate that magnetic resonance imaging (MRI) should be part of the primary standard work up of patients with rectal cancer because MRI can accurately identify the main risk factors for local recurrence and stratify patients into a differentiated treatment. The role of endoscopic ultrasound (EUS) is restricted to staging of superficial tumors because EUS is able to differentiate between T1 and T2 rectal cancer. Recent guidelines recommend the addition of diffusion-weighted (DWI) MRI to clinical and endoscopic assessment of response to preoperative radiochemotherapy (RCT). MRI is able to identify significant tumor regression which may alter the surgical approach.

## Zusammenfassung

Internationale Leitlinien empfehlen, dass die Magnetresonanztomographie (MRT) Teil der primären Standarduntersuchung von Patienten mit Rektumkarzinom sein sollte, da diese die Hauptrisikofaktoren für ein lokales Wiederauftreten genau identifizieren und die Patienten für eine differenzierte Behandlung stratifizieren kann. Die Rolle des endorektalen Ultraschalls (ERUS) beschränkt sich auf das Staging oberflächlicher Tumoren, da dieser in der Lage ist, zwischen T1- und T2-Rektumkarzinomen zu unterscheiden. Aktuelle Leitlinien empfehlen zudem die zusätzliche diffusionsgewichtete (DWI) MRT ergänzend zur klinischen und endoskopischen Beurteilung des Ansprechens auf eine präoperative Radiochemotherapie (RCT). Die MRT kann damit eine signifikante Tumorregression nachweisen, wodurch sich ggf. das chirurgische Vorgehen ändern kann.

## Local staging of rectal cancer

The problem after rectal cancer surgery has long been the high rate of local recurrence—up to 32%—due to incomplete resection of microscopic lateral spread of the tumor [1]. Although the impact of local recurrence on overall survival is not so great, its effect on quality of life is significant, with high morbidity due to severe pain, immobility, repeated chemotherapy, radiotherapy, and prolonged and multiple hospitalizations. Over the past decades, significant improvements were made in the management of these patients. Better surgery, preoperative radiotherapy (instead of postoperative), and the introduction of magnetic resonance imaging (MRI) have resulted in good local control with local recurrence rates well below 3%.

**Fig. 1:**
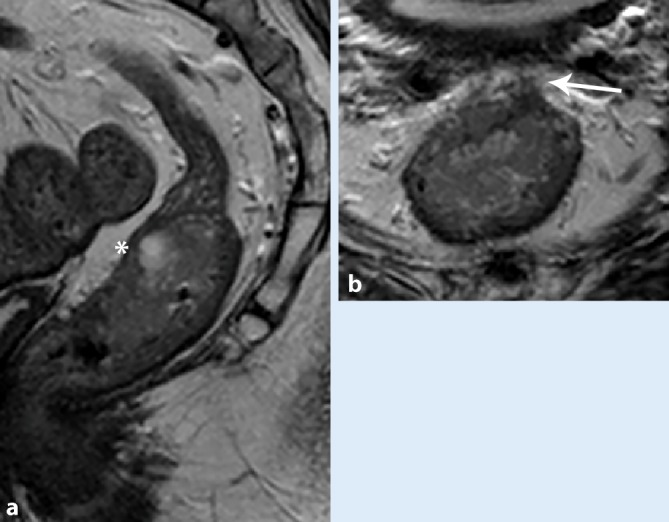
Sagittal and axial magnetic resonance images of a female patient with a T3ab tumor of the middle rectum (**a**; *white asterisk*) that penetrates the bowel wall on the anterior side (**b**; *black arrow*) but remains at a distance from the mesorectal fascia

The Dutch TME trial demonstrated that the risk for local recurrence differs among different groups of patients with rectal cancer [2]. On one side of the spectrum is the low-risk group: patients with superficial tumors that can effectively be treated with surgery (Fig. 1) or local excision (transanal resection). On the other side of the spectrum are patients at high risk for local recurrence. These patients have advanced tumors with either close relation to or involvement of the mesorectal fascia (MRF)—the circumferential resection margin of total mesorectal excision (TME)—or even extension into the surrounding organs. International guidelines [3] dictate that MRI should be part of the primary workup of patients with rectal cancer because it can reliably stratify treatment by identifying the risk factors for local recurrence [4]. In this article, the value of endorectal ultrasound (EUS) and MRI in rectal cancer staging and restaging will be discussed.

The MERCURY Study Group reported the results of a prospective trial with 408 consecutive patients on the value of MRI in assessing involvement of the MRF [5]. Eighty-seven percent of patients had a free resection margin (≥1 mm between the tumor and the tumor margin ). The sensitivity and specificity for assessment of a free resection margin were 59% and 92%, respectively. In 311 patients who had had no preoperative chemoradiation, the sensitivity for identifying an invaded resection margin was 42%, and the specificity was 98% [4].

In the meta-analysis of Lahaye et al., seven studies were included and showed a pooled sensitivity for MRI of between 60% and 88% for assessment of involved MRF [6]. The specificity varied between 73% and 100%.

For staging of superficial tumors, EUS is the preferred technique because all individual bowel wall layers are easily depicted in high resolution. EUS is the only imaging method that can differentiate between cT1 and cT2 tumors. It must be noted that the performance of EUS depends on the expertise of the sonographer [7], with expert sonographers generating better results than nonexperts. EUS is less accurate for staging advanced tumors, especially in assessing invasion of the tumor into the pelvic structures. Furthermore, EUS allows only a limited view of the entire mesorectum, and the TME resection margin and high stenosing tumors are often difficult to reach with the probe.

Bipat et al. published a meta-analysis on the value of EUS, computed tomography (CT), and MRI for T and N staging of rectal cancer [8]. Ninety studies, published between 1985 and 2002, were included The pooled sensitivity of EUS was 94% for the identification of T1–2, 90% for T3, and 70% for T4 stage tumors. The specificity was 86%, 75%, and 97%, respectively. The pooled sensitivity for identifying the T stage using MRI was 94% for T1–2, 82% for T3, and 74% for T4. The specificity for determining T1–2, T3, and T4 tumors with MRI was 69.5%, 76.5%, and 96%, respectively.

In the study of Bali et al., the diagnostic value of EUS (7 MHz) was investigated in 29 patients with rectal cancer [9]. It showed diagnostic accuracy of 79% for T staging, and for nodal staging it was around 60%.

Liersch et al. examined the value of EUS and CT in patients with advanced rectal carcinoma (T3, T4, and/or node-positive disease) [10]. In a subgroup that did not undergo preoperative treatment, staging was correct in 75% of the EUS patients and in 48% of the CT patients.

Panzironi et al. showed 80% sensitivity for EUS for the assessment of MRF invasion and 100% for both CT and MRI [11]. The sensitivity for T staging was 100%, 75%, and 92.3% for EUS, CT, and MRI, respectively, and for N staging it was 72.2%, 88%, and 76.4% for EUS, CT, and MRI, respectively. Peschaud et al. [12] reported sensitivity of 100% and specificity of 66% for the assessment of MRF invasion. The sensitivity for T staging was dependent on the stage and varied between 48% and 100%, with specificity between 68% and 91%.

Primary nodal staging by imaging remains very difficult. Sensitivities and specificities vary between 65% and 75% for MRI if size criteria are used. However, lymph node metastases of rectal carcinoma can be as small as a few millimeters, so no reliable size cutoff exists for malignant lymph nodes. It is known that when a lymph node is larger than 9 mm (short axis), the risk for malignancy is 93%. Lymph nodes as small as 2–5 mm have a 50% risk of malignancy. Other morphological characteristics, including irregular border, heterogeneous texture, and a round shape, are more predictive of malignancy than size is [13, 14]. In the international guidelines (European Society of Gastrointestinal and Abdominal Radiology [ESGAR] and Society of Abdominal Radiology guidelines), nodal staging therefore takes into account these morphological criteria [3]:

1. The absence of lymph nodes in the mesorectum is indicative of a cN0 status.
2. The presence of lymph nodes with a short-axis diameter >9 mm with or without malignant characteristics, such as irregular border, heterogeneous texture, or round shape, is highly predictive of a positive N status.
3. The presence of lymph nodes 5–9 mm in size with at least two of the criteria of irregular border, heterogeneous texture, and round shape is highly predictive of a positive N status.
4. The presence of lymph nodes smaller than 5 mm in size with all three criteria of irregular border, heterogeneous texture, and round shape is highly predictive of a positive N status.

## Restaging after chemoradiotherapy

**Fig. 2:**
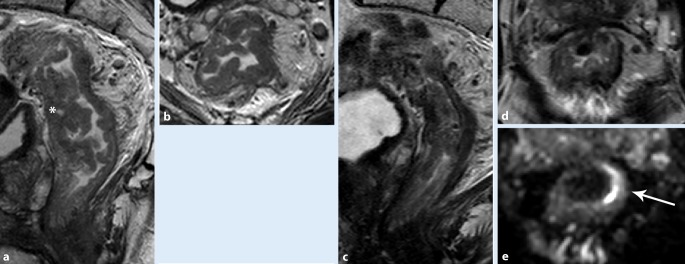
Magnetic resonance imaging (MRI) of a male patient before (**a**, **b**) and after chemoradiotherapy  (**c**, **d**) shows locally advanced rectal cancer (*white asterisk*) that responded poorly to the neoadjuvant chemoradiotherapy. Restaging MRI (**c**, **d**) shows a residual (isointense) mass (**e**; *white arrow*), and on diffusion-weighted imaging a restricted diffusion in the local tumor bed (**e**)

Restaging generally takes place 8–10 weeks after completion of chemoradiotherapy (CRT) and is performed with endoscopy and MRI. Restaging with MRI is recommended in the recent guidelines (Fig. 2; [15]). MRI can show tumor regression from an initially involved organ or mesorectal resection margin. This may alter the surgical approach; for example, if MRI shows significant regression of the tumor, TME can be considered instead of pelvic exenteration. Assessment of response is also relevant if nonoperative management (watch and wait) is considered for (near) complete responders [16].

**Fig. 3:**
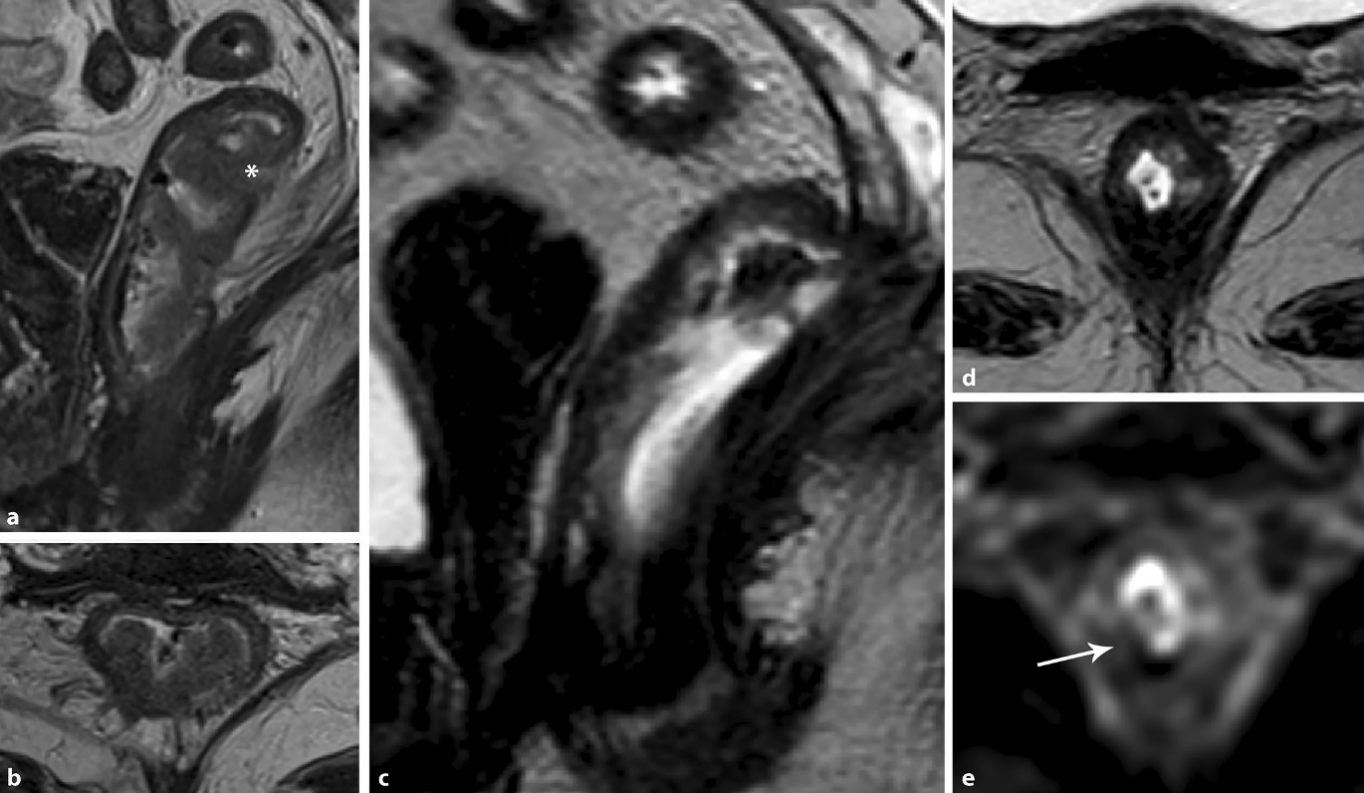
Magnetic resonance imaging (MRI) of a female patient before (**a**, **b**) and after chemoradiotherapy (**c**, **d**) shows a locally advanced distal rectal cancer (*white arrow*) that responded excellently to neoadjuvant chemoradiotherapy, with a complete response. On restaging MRI with diffusion-weighted imaging, no restricted diffusion was visible (**e**)

The meta-analysis by Van der Paardt et al. evaluated the diagnostic value of MRI for restaging of yTN and persistent involvement of the MRF after preoperative CRT [17]. It showed a pooled sensitivity of 76% and specificity of 86% for restaging of involved MRF. These results were confirmed in the meta-analysis by Huang et al. [18]. Both meta-analyses showed that MRI is insufficiently accurate, with a pooled sensitivity of 40.3% for the ypT stage and 19.1% for the ypT0 stage. However, the addition of diffusion-weighted imaging to MRI (DWI MRI) significantly improved the results, with a pooled sensitivity of up to 83.6% for the detection of ypT0 (pCR) ([17]; Fig. 3).

We found that endoscopy and digital rectal examination (area under the curve [AUC] 0.88) performed better than DWI and MRI (AUC 0.79) for identifying a ypT0 status. The highest accuracy, however, was achieved when combining the four assessment tools (AUC 0.91). For this reason, the International Watch & Wait Database (IWWD) Consortium stresses the importance of a multidisciplinary approach when selecting patients for watch-and-wait treatment [19].

Restaging of lymph nodes with MRI is more accurate than primary staging. After CRT the majority of lymph nodes decrease in size, and around 44% of the smaller lymph nodes (<4 mm) disappear. The absence of mesorectal and extramesorectal lymph nodes on restaging DWI MRI is highly predictive of a ycN0 status [20].

A recent multicenter analysis investigated 1216 patients with locally advanced rectal cancer treated with CRT followed by TME [21]. In 703 out of 968 patients, baseline MRI showed lateral pelvic lymph nodes. In 192 patients, these nodes were >7 mm. The study population had a 5-year local recurrence rate of 10%, of which half were lateral local recurrences (LLR). The group of patients with lateral nodes ≥7 mm at baseline MRI had a higher risk for LLR than the group with nodes <7 mm. The group that did not undergo lateral lymph node dissection (LLND) had a 19.5% rate of LLR at 5 years, versus 5.7% for the group that did undergo LLND. Hence, a 7-mm short-axis cutoff on MRI of lateral pelvic nodes seems to be a valuable predictor for LLR.

In conclusion, international guidelines dictate that MRI should be part of the primary standard workup of patients with rectal cancer because MRI can accurately identify the main risk factors for local recurrence and can stratify patients for differentiated treatment. The role of EUS is restricted to staging of superficial tumors because it is able to differentiate between T1 and T2 rectal cancer. The revised ESGAR guideline on rectal MRI includes a structured reporting template that is useful for clinical practice [15]. It provides the items that should be addressed in order to generate a comprehensive MRI report of rectal cancer. Recent guidelines also recommend the addition of DWI MRI to the clinical and endoscopic assessment of a patientʼs response to preoperative CRT. MRI is able to detect significant tumor regression, which may alter the surgical approach. Furthermore, DWI MRI combined with clinical and endoscopic examination can accurately identify patients with (near) complete response who could be considered for watch-and-wait nonoperative management.

## Practical conclusions

- MRI is mandatory in the primary workup of patients with rectal cancer and is recommended in the restaging workup after CRT.
- EUS is recommended in the initial staging workup of superficial tumors, and for medium-risk and higher-risk T1 tumors, subsequent MRI can be performed to assess the mesorectal nodal status.
- DWI sequences should be included in the restaging MRI protocol because DWI can accurately differentiate between residual disease and fibrosis after CRT.
- If organ-preserving treatment after CRT is considered, the combination of clinical examination, endoscopy, and DWI MRI is best for the assessment of a complete clinical response.
